# Production, purification and characterization of recombinant human R-spondin1 (RSPO1) protein stably expressed in human HEK293 cells

**DOI:** 10.1186/s12896-020-0600-0

**Published:** 2020-01-20

**Authors:** Gabriel Levin, Bruna Andrade Aguiar Koga, Gustavo Gross Belchior, Ana Claudia Oliveira Carreira, Mari Cleide Sogayar

**Affiliations:** 1 Cell and Molecular Therapy Center (NUCEL), Medical School, University of São Paulo, Edifício NUCEL, Rua Pangaré, 100 (Cidade Universitária), São Paulo, SP 05360-130 Brazil; 2 Department of Surgery, School of Veterinary Medicine and Animal Science, University of Sao Paulo, Sao Paulo, SP 13635-900 Brazil; 3 Biochemistry Department, Chemistry Institute, University of São Paulo, Sao Paulo, SP 05508-000 Brazil

**Keywords:** Tissue engineering, Heterologous mammalian protein expression system, Recombinant human R-spondin1 (RSPO1), Peptide growth factors, HEK293 cells

## Abstract

**Background:**

The R-Spondin proteins comprise a family of secreted proteins, known for their important roles in cell proliferation, differentiation and death, by inducing the Wnt pathway. Several studies have demonstrated the importance of RSPOs in regulation of a number of tissue-specific processes, namely: bone formation, skeletal muscle tissue development, proliferation of pancreatic β-cells and intestinal stem cells and even cancer. RSPO1 stands out among RSPOs molecules with respect to its potential therapeutic use, especially in the Regenerative Medicine field, due to its mitogenic activity in stem cells. Here, we generated a recombinant human RSPO1 (rhRSPO1) using the HEK293 cell line, obtaining a purified, characterized and biologically active protein product to be used in Cell Therapy. The hRSPO1 coding sequence was synthesized and subcloned into a mammalian cell expression vector. HEK293 cells were stably co-transfected with the recombinant expression vector containing the hRSPO1 coding sequence and a hygromycin resistance plasmid, selected for hygro^r^ and subjected to cell clones isolation.

**Results:**

rhRSPO1 was obtained, in the absence of serum, from culture supernatants of transfected HEK293 cells and purified using a novel purification strategy, involving two sequential chromatographic steps, namely: heparin affinity chromatography, followed by a molecular exclusion chromatography, designed to yield a high purity product. The purified protein was characterized by Western blotting, mass spectrometry and in vitro (C2C12 cells) and in vivo (BALB/c mice) biological activity assays, confirming the structural integrity and biological efficacy of this human cell expression system. Furthermore, rhRSPO1 glycosylation analysis allowed us to describe, for the first time, the glycan composition of this oligosaccharide chain, confirming the presence of an N-glycosylation in residue Asn137 of the polypeptide chain, as previously described. In addition, this analysis revealing the presence of glycan structures such as terminal sialic acid, N-acetylglucosamine and/or galactose.

**Conclusion:**

Therefore, a stable platform for the production and purification of recombinant hRSPO1 from HEK293 cells was generated, leading to the production of a purified, fully characterized and biologically active protein product to be applied in Tissue Engineering.

## Background

The R-Spondin (roof plate-specific Spondin) proteins comprise a family of secreted proteins, which are known for their important roles in cell proliferation, differentiation and death, by inducing the Wnt pathway [1, 2]. RSPOs are expressed in several embryonic tissues and in the adult, with adequate expression levels being essential for organism development and homeostasis maintenance [3, 4]. Several studies have demonstrated the importance of RSPOs in regulation of several tissue-specific processes, such as bone formation, skeletal muscle tissue development, pancreatic β-cells and intestinal stem cells proliferation and even cancer, as reviewed by Yoon and Lee [5]. However, inappropriate functioning of these proteins may lead to various pathological conditions [reviewed by [5, 6], such as: sexual phenotype reversal, hyperkeratosis and a predisposition for squamous cell carcinoma of the skin [7, 8], craniofacial defects and problems in limbs, lungs and hair follicles formation [9–12], in placental formation [13, 14] and in the development of nails (Anonychia) [15–18]. Therefore, it becomes evident that RSPOs display a great therapeutic potential for treatment of a number of diseases.

Four RSPOs proteins (RSPO1 through 4) have been described, all of which display characteristic domains which are conserved among vertebrates, such as: (1) a thrombospondin 1 repeat (TSR) domain, (2) a cysteine-rich furin-like (CR-FU) domain, (3) a basic amino acid-rich (BR) domain of variable length (carboxy-terminal region), and (4) a hydrophylic signal peptide (SP) sequence [5]. The signal peptide present at the protein amino-terminal region ensures its entry into the canonical secretory pathway, addressing to the endoplasmic reticulum, transit through the Golgi apparatus complex and secretion to the extracellular space [4, 19]. The RSPO CR-FU domain, in turn, is identified as being responsible for mediating the Wnt/β-catenin signaling pathway activation [4, 19–21], although other studies also suggest that this domain may be involved in regulating the secretion of these proteins [21]. On the other hand, the BR and TSR domains have been proposed to be responsible for regulating the intensity of RSPOs action in the canonical Wnt pathway induction [20]. In addition, the TSR and BR domains still seem to be responsible for the association of RSPOs to the extracellular matrix (ECM), by binding to glycosaminoglycans (GAG) and proteoglycans [4, 19, 22–24] [reviewed by [6]].

Currently, it is known that RSPO proteins are capable of inducing the canonical (beta-catenin-dependent) and non-canonical (beta-catenin-independent) Wnt pathways [reviewed by [5, 6]]. However, although several studies on RSPOs mechanisms of action are available, many questions still remain about their receptors and the mechanisms involved in signal transduction by these proteins. Studies revealed that RSPOs bind to leucine rich repeat containing G protein-coupled receptors 4–6 (Lgr4–6) [25–27] to induce the canonical Wnt/beta-catenin signaling pathway, but other studies also indicate that these proteins are able to bind to low-density lipoprotein receptor-related proteins 5–6 (Lrp5–6) [4, 19, 21, 28] and Kremen1 (KRM1) [29]. It is also known that RSPOs act by inhibiting the Zinc and Ring Finger 3 protein (ZNRF3) [30] and binding to Frizzled 8 (Fzd8) to induce the Wnt pathway [19], although, apparently, RSPOs only bind weakly to the FZD receptors [21, 28]. However, part of this controversy regarding RSPOs receptors may be explained by studies suggesting a synergistic action of these proteins with Wnt ligands [4, 31]. In addition, in a recent work, the non-equivalence of the WNT proteins and the RSPOs with respect to the induction of self-renewal in LGR5^+^ intestinal stem cells was observed, but cooperation between these proteins was highlighted [32]. Unlike the canonical Wnt pathway, the β-catenin-independent pathway seems to have a less contradictory status in the literature, although it is less explored and still presents several gaps. Recently, syndecans were found to be new RSPOs receptors in the Wnt pathway [24], but studies have shown that only RSPO2 and RSPO3 proteins bind to syndecans.

RSPO1 stands out among RSPOs molecules with respect to its potential therapeutic use, especially in the Regenerative Medicine field, due to its mitogenic activity in stem cells. This potential has been confirmed by several studies which have shown the use of RSPO1 in several animal models for treatment of: intestinal mucositis induced by chemotherapy [33] or radiation [34], inflammatory bowel diseases (IBD) such as ulcerative colitis (UC) and Crohn’s disease (CD), in which the inflammatory response leads to continuous intestinal epithelial cells death [31, 35] and Diabetes Mellitus (DM), as a cytoprotective and proliferative agent for β-cells, by regulating the canonical Wnt pathway [36, 37]. Furthermore, other studies suggest its use in joint diseases such as arthritis [38] and in cancer, possibly acting as a tumor suppressor gene in lymphocytic leukemia [38, 39].

The RSPO1 protein, produced and studied here, is comprised of 263 amino acid residues arranged in a single 28,959 Da polypeptide chain. According to the Universal Protein Resource database (UniProt), RSPO1 is encoded from the *RSPO1* gene, located on chromosome 1, position 38,076,951 to 38,100,595, presenting four isoforms, which arise from alternative exons splicing. In silico analysis of the RSPO1 protein demonstrated a three-dimensional structure rich in β-sheet secondary structures, lacking alpha helix. Recent studies demonstrated the presence of an N-glycosylation at the asparagine Asn137 of the polypeptide chain, which is related with RSPO1 secretion, activity and stability [40, 41], although both articles mentioned above present conflicting results about the effect of N-glycosylation on the biological activity of RSPO1 protein.

Here, the recombinant human RSPO1 was generated using a human cell line, namely: HEK293 (Human Embryonic Kidney) cells. Mammalian cells have been used for the production of several recombinant proteins, especially due to their ability to carry out post-translational modifications, which are essential for maintaining the structure and function of proteins. Among the post-translational modifications, glycosylation deserves special attention in the production of recombinant proteins in heterologous systems, since these modifications can interfere with protein folding, activity, stability and maturation, depending on the expression system used [42]. In this context, due to its capacity to generate complex glycosylation patterns, especially with the addition of sialic acids, the HEK293 cell line has been widely used for the production of recombinant proteins, being the human cell line most often used in the production of biopharmaceuticals approved by regulatory agencies, such as the FDA (Food and Drug Administration) [43, 44].

The objective of this study was to generate a stable expression platform for production of rhRSPO1 in human cells in order to obtain a purified, characterized and biologically active protein product. In the future, this platform may be optimized for rhRSPO1 production in an efficient and reproducible manner to be used in cell therapy. In addition to generation of rhRSPO1 overproducing cell clones, a new rhRSPO1 purification protocol has been established yielding a high purity protein product.

## Results

### Generation of the pNU1/RSPO1 construct

The optimized *RSPO1* DNA coding sequence was transferred from the pUC57 vector, in which it was synthesized, to the pNU1 expression vector, as shown in Additional file 1: Figure S1. The RSPO1-pNU1 construct generated was amplified in *E. coli* to be used in transfection of HEK293 cells. The DNA sequencing results indicated 100% identity with the optimized coding sequence of the *RSPO1* gene, confirming the cDNA integrity for transfection.

### Screening of HEK293 hRSPO1-producing cell clones

In order to select the rhRSPO1 overproducing cell clones, we isolated 37 HEK293 pNU1/RSPO1 cell clones, of which 10 were selected according to their growth capacity in culture. The selected clones were plated under two different conditions, namely: in the presence of fetal bovine serum (FBS) and in serum-free medium (SFM) and the conditioned media were collected for analysis after 48 h. Samples of the conditioned media were used in a Dot Blot immunoassay to compare the rhRSPO1 production levels by each cell clone under the same culturing and conditioning conditions, in order to select for the most productive cell clones for quantification of protein expression. The results of cell clones screening by Dot Blot demonstrated that several cell clones showed high rhRSPO1 expression levels in both FBS and SFM cultures.

Upon HEK293 cell clones screening by Dot Blot, two clones, named Cl.21 and Cl.L1, were selected for quantification of the rhRSPO1 produced, by ELISA, and for in vitro biological activity assays. The conditioned media collected from these clones maintained in the presence or absence of fetal bovine serum were diluted and assayed using the R-Spondin1 Human DuoSet ELISA kit. The results indicated a high level of rhRSPO1 production under both conditions, but slightly higher when cells were cultured in serum-containing medium. The HEK293-derived Cl.21 cell clone yielded a volumetric productivity of 1.25 μg/mL when grown in the presence of serum and 0.93 μg/mL under the serum-free condition, while clone L1 reached 1.94 μg/mL and 1.21 μg/mL, in the presence and absence of serum, respectively.

### Purification of rhRSPO1 from conditioned medium

The purification process of the rhRSPO1 protein produced in HEK293 cells consisted of a heparin affinity chromatography (Additional file 2: Figure S2), followed by molecular exclusion chromatography (Additional file 3: Figure S3). In the chromatogram of the first purification step, using a heparin column (Additional file 2: Figure S2A), it was possible to observe the presence of three absorbance peaks at the 280 nm UV wavelength, one at each NaCl plateau, indicating the release of proteins with different degrees of affinity to the column. In addition, the Western Blot assay (Additional file 2: Figure S2B) of the purification fractions, using a specific anti-RSPO1 monoclonal antibody, revealed that rhRSPO1 was preferentially released in the second step of NaCl concentration (713 mM), corresponding to the absorbance peak observed in this step. In the second purification step protocol, it was possible to observe that rhRSPO1 was released mainly in fractions A10 + A11, as may be observed in Additional file 3: Figure S3B.

**Fig. 1:**
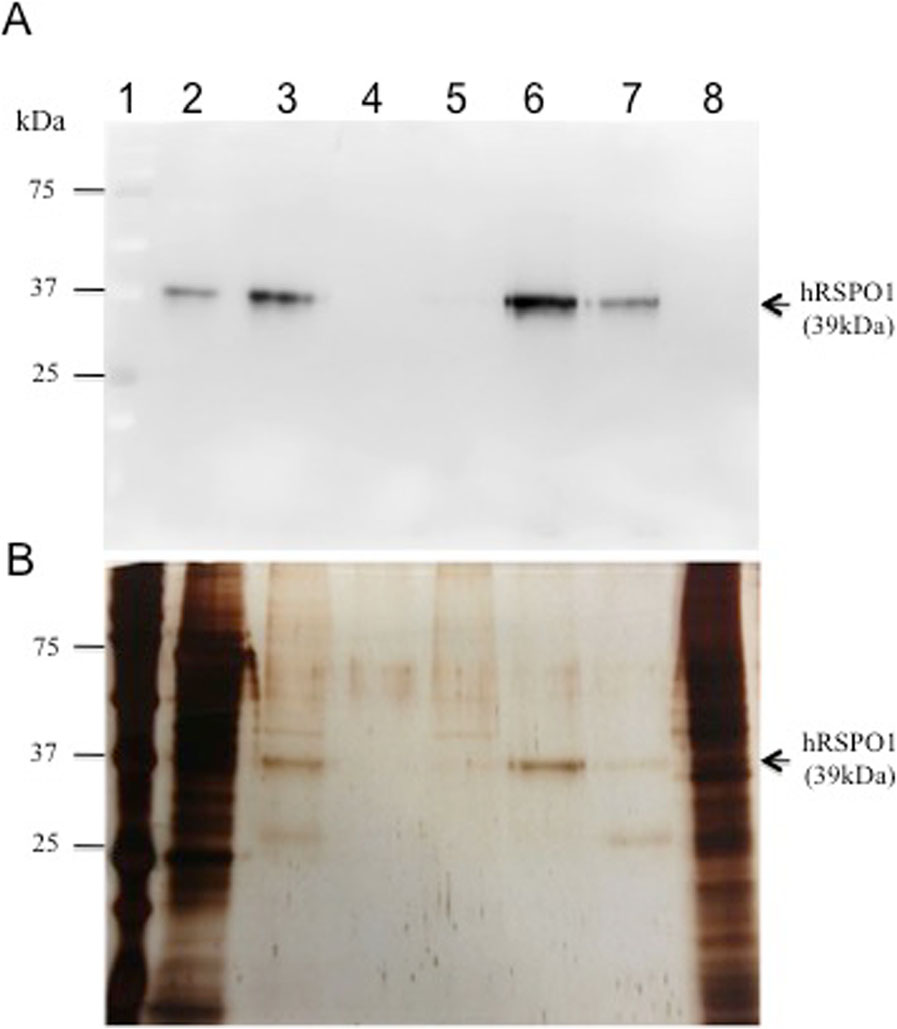
rhRSPO1 purification. **a** Western Blot analysis of purified rhRSPO1 using an anti-RSPO1 monoclonal antibody. **b** Silver-stained SDS-PAGE analysis of purified rhRSPO1. Samples: 1- Molecular weight marker; 2- Original conditioned medium from clone Cl.L1; 3- Step2 (after the Heparin Affinity column); 4- A8 fraction of the Molecular Exclusion column; 5- A9 fraction of the Molecular Exclusion column; 6- A10 fraction of the Molecular Exclusion column; 7- A11 fraction of the Molecular Exclusion column; 8- Conditioned medium from HEK293 cells transfected with the empty vector (negative control)

The rhRSPO1 degree of purity throughout the purification steps is shown in Fig. 1 and in Table 1. From the results presented, it is possible to observe a reduction of the presence of contaminating proteins, of different molecular weights, as the purification steps were implemented. The rhRSPO1 obtained from the pooled A10 + A11 fractions of the molecular exclusion column, run after the heparin affinity chromatography, showed a high degree of purity (90%), higher than the richest sample purified with only one chromatographic step (63%), and almost 30 times higher than the original conditioned medium (3.2%). The process used yielded a high purification efficiency with respect to the yield obtained after heparin affinity chromatography, with no loss in total rhRSPO1. However, despite the increased purity level, addition of the gel filtration step increased the loss of rhRSPO1 throughout the process, with a recovery rate of 50% (Table 1).

**Table 1:** Degree of purity and yield of the rhRSPO1 purification process

Samples	Efficiency (%)	Purity (%)
Original Conditioned Media	–	3.2
Heparin Affinity Column	100	63
Molecular Exclusion column	52	90

The efficiency of the purification process was calculated by dividing the total mass of rhRSPO1 in each fraction by the initial rhRSPO1 total mass used in the process. rhRSPO1 purity at each step of the purification process was assessed by silver-stained SDS-PAGE and subsequent densitometric analysis using the ImageJ software. The results are presented in percentages. Original Conditioned Medium (Cl.L1); Heparin Affinity Chromatography (Step2); Molecular Exclusion Chromatography (Fractions A10 + A11)

### rhRSPO1 in vitro biological activity

**Fig. 2:**
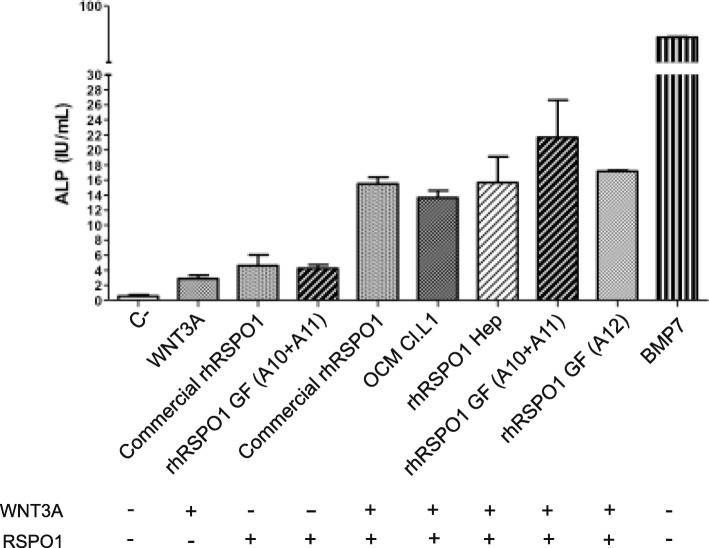
rhRSPO1 in vitro bioactivity. Alkaline phosphatase (ALP) colorimetric assay using a cell lysate obtained from C2C12 cells induced to osteogenic differentiation in the presence of the rhRSPO1 protein. Biological activity of purified rhRSPO1 - 100 ng/mL of rhRSPO1 from the original conditioned media or from various purification steps, and/or 100 ng/mL of WNT3A were used for induction of osteogenic differentiation. OCM: Cl. L1 original conditioned medium; Hep: rhRSPO1 from Step2 of the heparin column purification after buffer exchange; GF: rhRSPO1 from the second purification step using gel filtration (fractions A10 + A11 and A12). DMEM medium containing 5% FBS (C-) and medium conditioned by 293 T cells expressing human recombinant BMP7 were used as negative and positive controls, respectively. The commercially available rhRSPO1 protein (R&D Systems - Cod 4645-RS/CF) was used as reference sample for comparison (Commercial rhRSPO1). The (+) symbol indicates the presence of the listed recombinant protein, whereas traces (−) indicate its absence. ANOVA statistical test (Tukey’s post hoc test) was used and statistical differences were considered to be significant when the *p* < 0.05

To evaluate the rhRSPO1 in vitro biological activity, we used an osteogenic assay, with C2C12 cells to measure induction of alkaline phosphatase (ALP) activity. In vitro bioassays showed that the rhRSPO1 expressed by HEK293 cells displayed osteogenic activity (Fig. 2). As may be seen in Additional file 4: Figure S4, C2C12 cells treated with two doses of 200 ng/mL of rhRSPO1 obtained from all cell clones produced around 8 IU of ALP per milliliter of medium (significantly higher than the control), indicating the same protein specific activity, regardless of the cell clone.

C2C12 cells treated with the rhRSPO1 protein together with WNT3A displayed a significantly greater osteogenic activity than that of each of these agents used individually. Figure 2 shows that the purified rhRSPO1 protein produced by clone Cl.L1 maintained its biological activity in vitro and induced osteogenic activity in C2C12 cells, to levels similar to that presented by the commercially available rhRSPO1 protein, regardless of its purity level.

### rhRSPO1 in vivo biological activity

**Fig. 3:**
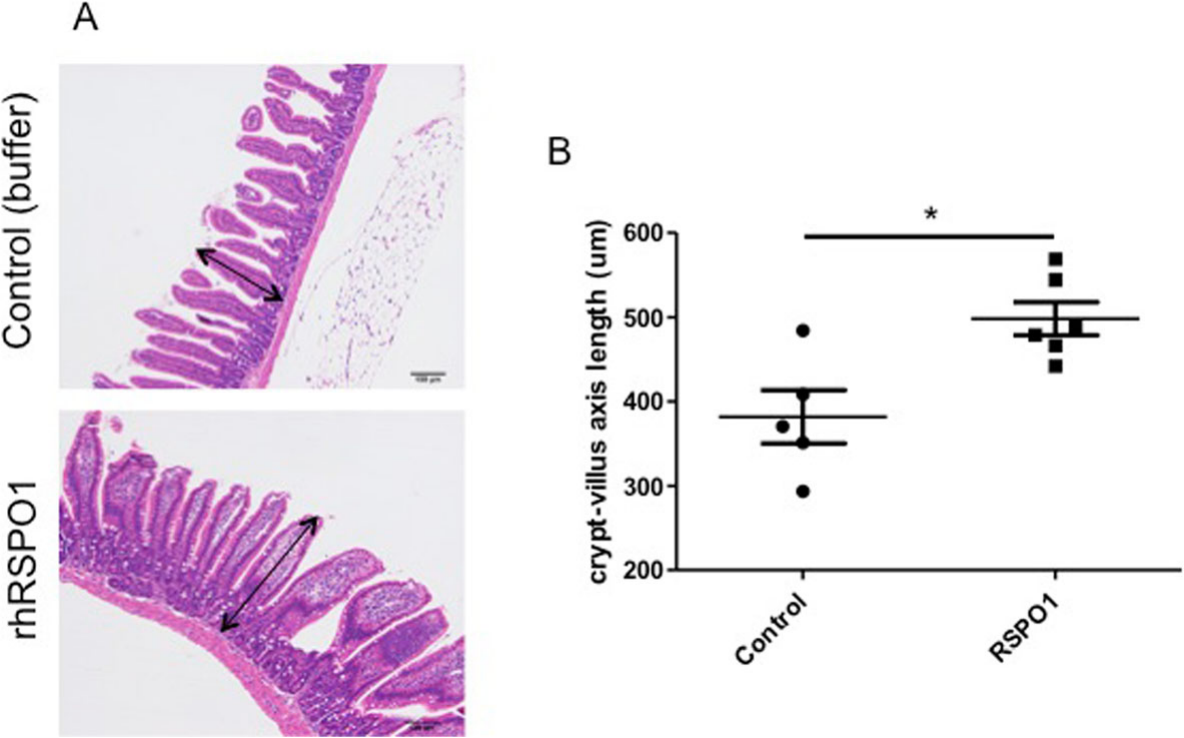
rhRSPO1 in vivo bioactivity. BALB/c mice were injected intravenously (i.v.) with 50 μg of rhRSPO1 (*N* = 7) or saline (40 mM Tris, 150 mM NaCl, 5% Trehalose), as a negative control (*N* = 5), for three consecutive days. After euthanasia, the mid-jejunum was collected and processed for histological analysis in order to evaluate the growth stimulatory effect of rhRSPO1. **a** Histological analysis (H&E). **b** Crypt-villus axis length. The arrows represent the crypt-villus axis and scale bar corresponds to 100 μm. Non-parametric student t-test (Mann-Whitney) was applied and statistical differences were considered to be significant when *p* < 0.05 (_*_)

In order to evaluate the in vivo biological activity, rhRSPO1 was injected intravenously in BALB/c mice, as described in the Methods section. After treatment for three consecutive days, histological analysis demonstrated a significant difference between the rhRSPO1 (498.4 ± 19.76 μm) and control groups (381.7 ± 31.65 μm), with respect to the mean crypt-villus length of the mid-jejunum region of the small intestine (Fig. 3), suggesting that treatment with rhRSPO1 induced growth of the intestinal epithelium in this animal model. In addition, macroscopic analysis of the diameter of the mid-jejunum of the rhRSPO1-treated animals tended to increase, when compared to the animals which received only saline solution, however, this difference was not statistically significant, possibly by a limitation of the technique employed in the measure (data not shown). All animals listed in the methodology were included in the analysis of the results.

### Structural characterization of rhRSPO1

#### PNGase F deglycosylation assay

**Fig. 4:**
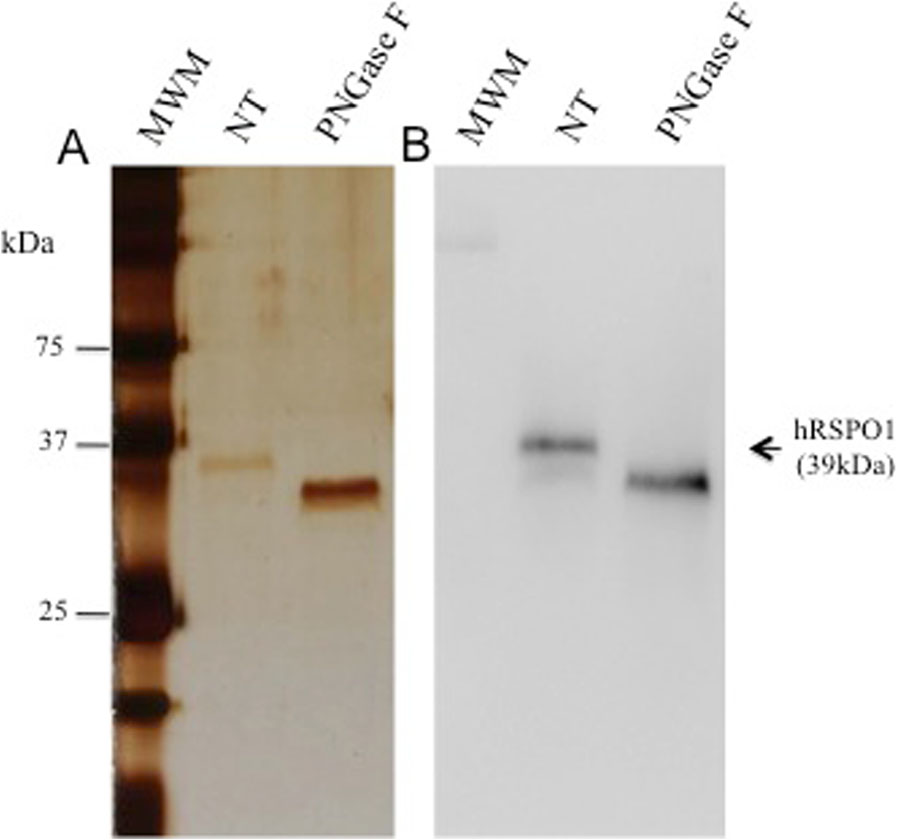
Deglycosylation of rhRSPO1 with PNGase F**.** For each sample 100 ng of purified rhRSPO1 (A10 + A11 fractions from the molecular exclusion chromatography) was applied, under denaturing conditions, previously deglycosylated with N-glycosidase F (PNGase F) or without treatment (NT). **a** Silver-stained SDS-PAGE analysis of purified rhRSPO1. **b** Western blot analysis of purified rhRSPO1 using an anti-RSPO1 monoclonal antibody. MWM: Molecular weight marker

As may be seen in Fig. 4, digestion of the rhRSPO1 protein with N-glycosidase F (PNGase F) resulted in an increase in the electrophoretic migration of the protein under denaturing conditions, indicating that the rhRSPO1 produced in this work exhibits N-glycosylation.

#### Lectin panel assay

Analysis of rhRSPO1 protein using the Lectin Panel kit showed a glycosylation profile which includes terminal sialylation at the O- or N-linked residues, as indicated by binding of SNA (*Sambucus nigra* agglutinin) and MAA (*Maackia amurensis* agglutinin) lectins. SNA (relative value 0.15) indicates the presence of sialic acid linked (2–6 bond) to galactose, while MAA indicates the presence of sialic acid linked (2–3 bond) to galactose (relative value 0.12). Detection of DSA (*Datura stramonium* agglutinin - relative value 0.17) indicates that some molecules in the sample also had either N- or O-linked Gal (1–4) -GlcNAc glycan structures and/or GlcNAc as an O-linked residue. The GNA lectin (*Galanthus nivalis* agglutinin) was identified in the sample, however, the result is inconclusive to determine the presence of terminal mannose, since the signal for this lectin was also detected in the elution buffer used as the negative control. From this result (Additional file 5: Figure S5), it is possible to infer that the rhRSPO1 generated in our laboratory has hybrid glycan structures containing terminal sialic acid, N-acetylglycosamine and/or galactose residues.

#### LC-MS/MS analysis

Protein analysis by liquid chromatography (LC), followed by mass spectrometry (MS), showed that the rhRSPO1 protein produced has a molecular mass of 28.94 kDa. The LC-MS/MS analysis allowed additional information on the glycosylation pattern of the protein, confirming the location of a glycosylation site on the Asp137 by the presence of a 162 Da mass (Additional file 6: Figure S6). This mass was consistently found in the spectrum indicating the presence of glycosylation (glycation). The mass of an O-GlcNAc would be 203 Da, which although predicted was not found.

## Discussion

Here, we describe the production, purification and characterization of recombinant human RSPO1 expressed in HEK293 cells, which is the human cell expression system most commonly used for the production of biopharmaceuticals. The rhRSPO1 protein produced was purified, characterized with respect to its peptide structure and glycosylation pattern, in addition to in vitro and in vivo biological activity. During this process, a novel purification strategy, using two sequential chromatographic steps, was designed in order to optimize the production of the target protein with high efficiency and purity.

The results presented here indicate that HEK293 cells expression system used was efficient for rhRSPO1 production, leading to good levels of RSPO1 production and biological activity. The rhRSPO1 protein could be detected in the cell culture medium through the Dot Blot, Western Blot and ELISA assays, thus confirming its secretion into the extracellular medium. This is consistent with the presence of a putative signal peptide sequence, corroborating some literature reports describing the secretion of RSPOs to the extracellular medium [4, 19]. According to Nam et al., the subcellular localization of RSPO proteins in the endoplasmic reticulum and in the Golgi complex indicates that RSPOs are processed through the canonical secretory pathway [19]. However, unlike previous studies describing low levels of RSPOs in the conditioned medium, possibly due to the protein association to the extracellular matrix (ECM), the cell surface, or both [4, 19], in the present work, the rhRSPO1 protein produced in human HEK293 cells reached high levels, with some overproducing clones, such as Cl. L1, reaching 1.94 μg/mL and 1.21 μg/mL in conditioned medium, maintained in the presence or absence of FBS, respectively.

Heparan sulfate proteoglycans (HSPGs), including Sindecans and Glypicans, are located on the cell surface and in the ECM, functioning as regulators of various cell signaling pathways, including those of WNT, FGF, BMP, and SHH [45, 46]. According to the literature, treatment of cells with soluble heparin or sodium chlorate, an inhibitor of GAGs sulphation, significantly increases the level of free RSPO protein detected in the conditioned medium and strongly bound to immobilized heparin [19], suggesting that RSPOs may bind to HSPGs. However, since the RSPO1 produced in the present work was detected at high levels in the conditioned medium, other strategies to increase the release of this protein in the conditioned medium were not necessary, thus avoiding the use of additives to the culture medium. Furthermore, although domain deletion studies have demonstrated that RSPO proteins lacking the BR domain or both the TSR and BR domains were easily detected as soluble proteins in the conditioned medium, with significantly lower affinity to heparin [4, 19], another study found that RSPO proteins lacking these domains activate the canonical WNT signaling pathway less effectively [20]. Therefore, production of the entire rhRSPO1 protein, with its complete structure, was chosen, without any domain deletion.

In addition, the rhRSPO1 protein, produced and purified in this work, was shown to be biologically active, displaying an in vitro activity similar to that of the commercially available rhRSPO1 (R&D Systems), as demonstrated by alkaline phosphatase assay following osteogenic induction of C2C12 cells. Consistent with the previously described synergistic action of RSPOs and WNT ligands, addition of Wnt3A during rhRSPO1 induction of C2C12 cells led to increased alkaline phosphatase produced levels relative to those verified upon treatment with the rhRSPO1 protein alone, indicating that osteoblastic differentiation activity was more pronounced [31, 47]. In addition, the in vivo biological activity assay upon injecting (i.v.) mice with 50μg of rhRSPO1 for three consecutive days demonstrated that this protein also has biological activity in this model. An increased diameter of the small intestine (mid-jejunum) of the animals was observed upon histological analysis of the tissues, although this difference was not seen macroscopically. This analysis demonstrated that the height of the crypt-villus axis was significantly higher in the rhRSPO1-treated animals compared to the control group, suggesting that rhRSPO1 induced intestinal epithelial growth, probably by inducing intestinal stem-cells (LGR5^+^) proliferation [31, 48–50]. However, it is noteworthy that despite the significant inductive effect of rhRSPO1, this protein was not able to duplicate the intestinal diameter in the animals, as reported by Kim and coworkers [31] in this same model, possibly due to variability among animals or technical accuracy of measurements.

Regarding the purification of rhRSPO1, it is worth mentioning that we developed a new purification strategy using two sequential chromatographic steps, allowing us to optimize the production of the target protein in a stable manner and with a high purity level (90%). Throughout the process, there was an increase in the purity level of the target protein at each chromatographic step, resulting in almost 30 times enrichment relative to the initial sample. Firstly, affinity chromatography allowed the separation of the proteins with heparin binding domains, as in the case of RSPO1, from the others, and then the molecular exclusion chromatography allowed separation of the proteins by size. Previously, three main articles in the literature briefly described the production of recombinant human RSPO1 protein in mammalian cells [31, 35, 51]. Kim & cols used HEK293 cells adapted to growth in suspension, as a biological system for expression of rhRSPO1, obtaining protein levels around 2.5 mg/L, with high purity [31]. In parallel, Zhao and colleagues expressed the recombinant RSPO1 in the CHO (Chinese Hamster Ovary) cell line, also reaching a high purity level, however, details on protein expression were not revealed [35]. In both of these articles, ion exchange chromatography was used for rhRSPO1 purification. On the other hand, Tsuchiya et al., used the HT1080 human fibrosarcoma cell line to express rhRSPO1 and Heparin Sepharose 6 beads for protein purification, however, details of the process, as well as the purity level reached, were not described [51].

Two other groups also expressed fragments of RSPO1 in systems other than mammalian cells. Moad and Pioszak used *E. coli* bacteria cells to stably express the cysteine-rich furin-like domains 1–2 (CR-FU1–2) of the RSPO1 protein, proposing this methodology as a low-cost form of production [52]. Wang and colleagues, in turn, also expressed and purified a human RSPO1 fragment containing the CR-FU1–2 domains, using baculovirus-infected insect cells [53]. However, as previously mentioned, although some authors claim that the CR-FU domain is sufficient to potentiate the WNT pathway [4], another study states that proteins without the BR and TSR domains are less efficient to activate the canonical WNT pathway [20].

The structural characterization analyses of rhRSPO1 produced in the present work allowed us to add important information to those already present in the literature, describing, for the first time, the glycan composition of its oligosaccharide chain. From the LC-MS/MS glycosylation assay performed, it was possible to confirm the presence of an N-glycosylation in Asn137 of the polypeptide chain, as described by other authors [40, 41], by the presence of 162 Da mass in the spectrum. Although the LC-MS/MS analysis led to identification of glycosylation (glycation), a broader analysis, including the results of PNGase F digestion and the lectin panel, points to the presence of a more complex hybrid glycan structure containing terminal sialic acid, N-acetylglucosamine and/or galactose. The fact that other types of glycosylation were not found by LC-MS/MS analysis does not rule out the possibility of such structures in the sample, since fragments with multiple glycosylation events are often protected from enzymatic digestion and do not generate ionized fragments, especially due to the hydrophobic nature of the glycosylation-containing fragment. Therefore, the lectin panel results and the PNGase F digestion complement the LC-MS/MS analysis, generating a more robust description of the rhRSPO1 glycosylation profile.

N-linked glycosylation plays a crucial role during maturation and secretion of many proteins [54]. From assays with deglycosylated mutant proteins, Chang et al. (2016) concluded that the presence of N-glycosylation in Asp137 positively interferes with rhRSPO1 protein secretion, intracellular stability and slightly with the secreted protein stability, being important for its accumulation in the culture medium [41]. The authors further suggest that glycosylation is important for rhRSPO1 folding and does not interfere with its heparin binding ability. Conversely, in 2017, Tsuchiya and colleagues demonstrated that N-glycosylation reduced rhRSPO1 secretion to the culture medium and, also, its ability to induce the canonical Wnt pathway [51]. The conflicting results presented by these two groups highlight the importance of characterizing the rhRSPO1 glycosylation profile, since the recombinant proteins used in each of these articles were generated using different expression systems and possibly present differences in their glycosylation profile.

Results from the literature suggest that glyco-engineering can be used to improve the therapeutic function of recombinant proteins. Addition of terminal sialic acids to proteins glycan chains, as observed in the rhRSPO1 produced here, positively contributes to greater stability and half-life of different proteins [55], and may also reduce possible immune reactions [56]. Therefore, characterization of rhRSPO1 glycosylation carried out in the present work further advances the attempt to conciliate the differences regarding the role of rhRSPO1 glycosylation in its folding, secretion, stability and biological activity, depending on the protein expression system used. It is noteworthy that the proteins produced in HEK cells display high similarity to those naturally occurring in humans in terms of post-translational modifications and function, justifying the choice of this system to express rhRSPO1 [57]. However, further experiments are necessary to complete the glycans characterization of rhRSPO1 and to conciliate the divergent results regarding the effect of N-glycosylation on the activity of the protein presented by the two groups mentioned above and, also, to establish any possible correlation between rhRSPO1 glycosylation and its secretion, stability and biological activity.

As previously mentioned, all four RSPOs possess a TSR1 domain which binds to heparin or HSPGs [19]. C-mannosylation and O-fucosylation were indicated as domain-specific glycosylation forms of the thrombospondin type 1 module [58]. Although predicted, the 203 Da mass relative to an O-GlcNac was not found in the rhRSPO1 by LC-MS/MS analysis. Mass spectrometric analysis also provided information on the mass of rhRSPO1, which is 29 kDa, as present in the databases. However, an electrophoretic migration of RSPO1 protein consistent with a molecular mass of approximately 39 kDa was observed through SDS-polyacrylamide gel electrophoresis (SDS-PAGE) under reducing and denaturing condition, as reported by others in the literature, possibly due to either changes in the three-dimensional conformation pattern of the protein or to glycosylation.

As previously highlighted, the RSPO1 protein has great therapeutic potential in the field of Regenerative Medicine, especially due to its mitogenic activity in stem cells. According to Abo & Clevers, the use of WNT pathway modulators, such as RSPO proteins may be a clinically safer option than the use of WNT ligands, in view of their ability to regenerate injured tissue without violating the natural order of events of tissue regeneration [59]. Therefore, unlike WNT ligands, which have a pleiotropic effect in the body, RSPO1 could be used to potentiate tissue repair of specific tissues, avoiding possible side effects on other tissues of the body, since induction of the WNT pathway by these proteins is tissue-restricted. In addition, the non-equivalence of the WNT and RSPOs proteins relative to induction of self-renewal in LGR5^+^ intestinal stem cells was observed in a recently published work [32], highlighting the cooperation that takes place between these proteins. Therefore, the methodologies here described for production and purification of a biologically active recombinant human RSPO1 to be used in Cell Therapy open new avenues for further optimization of its bioprocess and may contribute, in the future, to treatment of medical conditions, such as chemotherapy [33] or radiation-induced mucositis [34], inflammatory bowel diseases [31, 35], diabetes [36, 37], joint diseases [38] and even cancer [38, 60], as previously suggested by several authors based on animal models.

## Conclusions

In this work, a stable platform for production of recombinant hRSPO1 in HEK293 cells was generated, as well as an efficient and reproducible protocol for purification of this protein, thus allowing the production of a purified, fully characterized and biologically active protein product to be applied in Tissue Engineering and investigative studies. The present work paves the way for further optimization of the rhRSPO1 bioprocess aiming at its biopharmaceutical application in the future. In addition, the structural characterization of rhRSPO1 peptide chain and of its glycosylation profile, identifying glycan structures such as terminal sialic acid, N-acetylglucosamine and/or galactose, should contribute to better understanding of this protein in future, regarding its activity, stability and other important factors for its clinical and research use. However, further studies are still required to fully understand the molecular mechanisms involved in RSPO1 activity, secretion, stability and other aspects involved in tissue repair.

## Methods

### Synthesis of the coding sequence of the human *RSPO1* gene

The 791 bp cDNA sequence encoding the *RSPO1* variant 1, deposited in the National Center for Biotechnology Information database (NCBI - NM_001038633), was synthesized by GenScript USA Inc. Company). The synthesized nucleotide sequence is the result of modification of the original sequence encoding the *RSPO1* gene, upon a codon optimization process, according to the OptimumGene™ algorithm, in order to optimize the efficiency of gene expression in CHO cells, retaining the integrity of the protein sequence. The synthesized cDNA product was cloned into the pUC57 vector.

### Plasmid vectors

The pNU1 bicistronic plasmid expression vector was developed in our laboratory from the pIQID vector [61], with the insertion of a multiple cloning site (MCS) and removal of the Gateway® recombination elements. The following main elements are present in the pNU1 vector: a- PolyA signal; b- Internal ribosome entry site (IRES); c- Chicken beta-actin promoter; d- CMV IE enhancer; e- mDHFR CDS; f- Col E1 Ori; g- Amp^r^; h- MCS.

The pX343 selection plasmid vector, derived from the pY3 vector [62], presents the bacterial hygromycin B resistance gene under control of the Mo-MuSV LTR cloned into pBR322 [63].

### Sub-cloning of the optimized *RSPO1* cDNA insert into the pNU1 expression vector for mammalian cells and selection of bacterial clones

The pNU1 mammalian expression vector, designed and constructed by our group, was digested with the *Eco*RI and *Not*I restriction enzymes. The coding sequence corresponding to the human *RSPO1* synthesized product was also digested with the same enzymes in order to release the insert of interest and use it for vector exchange. After separation and purification, the digestion products were subjected to a ligation reaction, catalyzed by the T4 DNA Ligase (Thermo Fisher Scientific Inc.) enzyme and the product of this reaction was used to transform *E. coli* XL1 Blue MRF bacteria by electroporation. Positive clones selected in culture medium containing the antibiotic to which the vector confers resistance (ampicillin) were selected upon PCR reactions for amplification of the coding sequence of the *RSPO1* gene. Positive bacterial clones were cultured in LB culture medium in the presence of the antibiotic (ampicillin) for plasmid DNA preparation of the pNU1/hRSPO1 construct. The plasmid product obtained was subjected to DNA sequencing reactions, by the Sanger method, using the BigDye Terminator v3.1 Cycle Sequencing Kit (Thermo Fisher Scientific Inc.).

### Cell line and culture conditions

HEK293 cells were purchased from the American Type Culture Collection (ATCC- ATCC® Number: CRL-1573™) and cultured in adherent flasks in Dulbecco’s Modified Eagle’s Medium (DMEM) supplemented with 10% Fetal Bovine Serum (FBS), 1 mM sodium pyruvate, 1.2 g/L sodium bicarbonate, 25 mg/L ampicillin and 100 mg/L streptomycin, in a humidified atmosphere containing 2% CO_2_–98% air, at 37 °C. To ensure microbiological control of the process, all cell strains were tested for Mycoplasma by analysis of polymerase chain reaction (PCR), using specific primers, as previously described by Uemori and colleagues [64].

### Transfection of HEK293 cell lines and cell clones isolation

HEK293 cells were stably co-transfected with the pNU1/hRSPO1 plasmid construct, together with the pX343 hygromycin B resistance plasmid, using a 40:1 ratio, respectively, using liposomes (Lipofectamine® 2000, Thermo Fisher Scientific Inc.). 48 h later, the transformant cells were selected in medium containing 100 μg/mL of hygromycin B and, after an additional 48 h period, cells were diluted and maintained in selective medium (DMEM 10% FBS containing 100 μg/mL of hygromycin B) for growth of colonies derived from a single cell, for isolation of cell clones through colony hand picking using cloning stainless steel cylinders. After HEK293 cell clones isolation, the cultures were maintained in adherent flasks, under the same conditions described above.

### Analysis of the rhRSPO1 protein expression

The levels of rhRSPO1 protein released into the conditioned medium by each cell clone were analyzed using a specific human RSPO1 ELISA kit (Human R-Spondin 1 DuoSet ELISA – R&D), Dot Blot and Western Blot immunoassays. A monoclonal antibody against hRSPO1 (ab81600 - Abcam) was used for immunoblots. For HEK293 cells, the culture medium was conditioned during 48 h by each cell clone (10^6^ cells) plated in 60 mm plate with 10% Fetal Bovine Serum (FBS) or Serum-Free Media (SFM) and used in the assays. Medium conditioned by HEK293 cells transfected with the pNU1 empty vector (pNU1Ø) was used as the negative control.

### Purification of rhRSPO1 from conditioned media

The recombinant human RSPO1 protein was purified using the fast protein liquid chromatography (FPLC) Äkta Purifier system UPC-100 (GE Healthcare). To this end, an affinity chromatography was performed using a HiTrap™ Heparin HP column, followed by a molecular exclusion chromatography step using the Superdex 75 10/300 GL column (GE Healthcare). The purified protein was characterized by Western Blot and quantified by ELISA. The protein purity was assessed by silver-stained SDS-PAGE and subsequent densitometric analysis using the ImageJ software. For the first rhRSPO1 purification step by affinity chromatography, a 5 mL heparin column was used. Filtered and degassed buffers were also used in the process, namely: equilibration (40 mM Tris with 4 M Urea, pH 7.4) and elution (40 mM Tris, with 1 M NaCl and 4 M Urea, pH 7.4) buffers. Throughout the process, a constant flow rate of 3 mL/min was used, respecting the pressure limit of 0.7 MPa. Before sample application, the column was prepared with two column volumes (CV) of equilibration buffer (40 mM Tris with 4 M Urea) and then the conditioned medium sample was loaded into the system. After sample application, the column was washed with 5xCV to remove the non-bound proteins and then the bounded proteins were eluted in a NaCl segmented gradient with three steps, namely: step 1- 356 mM; step 2- 713 mM; and step 3- 1 M NaCl segment. After purification, the column was washed with 2xCV of 40 mM Tris-Cl buffer containing 2 M NaCl and re-equilibrated with 5xCV of equilibration buffer.

For the second purification step, using a molecular exclusion column, a 40 mM Tris-HCl buffer, containing 713 mM NaCl and 5% Trehalose, pH 7.4, appropriately filtered in 0.22 μm membrane and degassed, was used. Prior to the run, the column was equilibrated with 2xCV of buffer and then loaded with the sample coming from the heparin column purification. The applied sample was previously concentrated to 300 μL, using ultrafiltration with a 10 kDa cut-off filter. Throughout the process, a constant flow rate of 0.5 mL/min was used, respecting the pressure limit of 1.8 MPa and the eluates were collected.

### rhRSPO1 in vitro biological activity

The biological activity of rhRSPO1 was tested in vitro by a colorimetric alkaline phosphatase activity assay (ALP), using C2C12 cells, which undergo osteoblastic differentiation mediated by activation of the Wnt/β-catenin, upon treatment with rhRSPO1, a method adapted from that described by Lu and colls [47]. C2C12 cells were plated at low density in DMEM supplemented with 10% FBS and grown for 48 h until reaching 80% confluence. On day zero of the treatment, the culture medium was changed to DMEM 5% FBS containing 100 ng/mL of rhRSPO1, either individually or in combination with 100 ng/mL of rhWNT3A (R&D- Cod 5036-WN) [31, 47] and, 2 days later, the cells were induced again with the same protein dose. On the 5th day of treatment, cells were harvested, lysed, using a lysis buffer (0.5 M Tris-Cl pH 9.0, 0.9% NaCl, 1% Triton X-100) and the samples were centrifuged at 12,000 g for 15 min. The ALP activity test was carried out according to the ALP colorimetric assay kit manufacturer’s instructions (Labtest) in an assay adapted for 96-well plates. Subsequently, 10 μL of cell lysate was mixed with 50 mL of reaction buffer (150 mM NaCl, pH 10.1), and the mixture was incubated at 37 °C for 5 min. The reaction was stopped by adding 200 μL of color reagent (94 mM sodium citrate, 250 mM NaOH), and then used for absorbance reading at 590 nm. Conditioned medium from HEK293 cells transfected with the pNU1 empty vector or DMEM 5% FBS were used as negative controls. Conditioned medium from 293 T cells expressing the recombinant human Bone Morphogenetic Protein 7 (rhBMP7) was used as positive control [65] and the commercial rhRSPO1 protein (R&D Systems - Cod 4645-RS/CF) was used as reference sample for comparison.

Prior to use, the rhRSPO1 sample from step 2 of heparin affinity chromatography purification underwent a buffer exchange to urea removal, using ultrafiltration with 10 kDa cut-off column. Forty mM Tris-HCl buffer, containing 713 mM NaCl and 5% Trehalose, pH 7.4, was used in buffer exchange, in a volume amount equal to 10 times the sample volume. For the assay all samples were filtered on 0.22 μm filter (MILLEX GP Filter Unit – Millipore Express PES Membrane) for sterilization.

### rhRSPO1 in vivo biological activity

All animal experimentation was approved by the Ethics Committee for Animal Use (CEUA) of the Medical School on 08/26/2015 (protocol n° 131/15), University of São Paulo, São Paulo, Brazil, in accordance with the National Council for the Control of Animal Experimentation (CONCEA).

The wild type BALB/c mice used in this study were provided by the SPF animal facility of the University of São Paulo Medical School and kept in the experimental animal facility during the experiment period. All animals used in the study were male, adults (10–12 weeks) weighing 22-29 g (mean = 25.2 g), kept in groups of up to 6 animals in ventilated cages (Alesco) bedded with wood shavings, with environmental enrichment, at 22 °C and controlled humidity, defined light/dark cycles (12/12 h), receiving filtered autoclaved water and irradiated Nuvital food ad libitum.

The biological activity of rhRSPO1 was tested in vivo using BALB/c mice as a model, as described by Kim et al. [31]. Fifty microgram of rhRSPO1 (*N* = 7) or saline buffer (40 mM Tris, 150 mM NaCl, 5% Trehalose), as a negative control (*N* = 5), was injected intravenously (i.v.) into the BALB/c mice for three consecutive days in the morning. The animals were randomly selected for allocation in the experimental groups. Samples were applied by retro-orbital injection using individual and disposable insulin needle (0.3 mm) in a volume of 200 μL per application. Prior to application, the samples were concentrated and buffer exchanged (40 mM Tris, 150 mM NaCl, 5% Trehalose) using 10 kDa cut-off column for salt reduction. After treatment, the animals were euthanized in a CO_2_ chamber, dissected and the small bowel was collected for measurement of the mid-jejunum diameter to evaluate the stimulatory effect of rhRSPO1 on intestinal growth. Furthermore, histological analysis was carried out in order to assess the effect of rhRSPO1 treatment on the organ.

For histological analysis, the samples were collected and fixed in 4% paraformaldehyde. After fixation the tissues was washed in phosphate buffered saline (PBS), dehydrated in increasing concentrations of ethanol, diaphanized in xylene and embedded in Histosec embedding agent (Merck Millipore). The paraffin-like-embedded tissues were sectioned into 5 μm-thick sections using an automatic microtome (Leica - RM2165), placed onto histological slides and stained with hematoxylin-eosin (H&E). The slides were recorded with the NIS-Elements software on a Nikon 80i microscope (CADI-FMVZ) and image analysis was performed using ImageJ software. To determine the mean length of the crypt-villus axis of the mid-jejunum region after the treatments (continuous quantitative variable), the length of the base of the crypt up to the apex of the villus was measured for as many possible intact crypt-villus pairs and the mean values obtained for each animal were used as a biological replicate.

### PNGase F Deglycosylation assay

To verify the presence of N-glycosylation in the purified rhRSPO1 protein produced in this work, this protein was subjected to digestion with the PNGase F enzyme (New England Biolabs) under denaturing conditions according to the protocol provided by the manufacturer. One microgram of rhRSPO1 was incubated in Glycoprotein Denaturing Buffer (0.5% SDS, 40 mM DTT) at 100 °C for 10 min. After denaturation, 1 Unit of PNGase F was added to the sample in 1X Reaction Buffer (50 mM sodium phosphate, 1% NP-40, pH 7.5), and the reaction was incubated at 37 °C for 1 h. One hundred nanogram of PNGase F digested sample or without treatment were fractionated on a 15% SDS-PAGE and analyzed by Silver staining and Western Blot methods, as previously described.

### Lectin ELISA assay

To obtain a glycosylation qualitative profile of the recombinant human RSPO1 protein, a lectin panel assay containing different lectins was employed with the DIG glycan differentiation kit (ROCHE), according to the protocol adapted from Legardinier et al. [66].

ELISA plate (NUNC-Immuno Plate/ MaxiSorp-NUNC) was coated with 200 ng/μL of purified rhRSPO1 and standard glycoproteins (carboxypeptidase, transferrin, fetuin, asylophetuin), as controls. Specific types of glycan residues in the rhRSPO1 molecule were detected by different lectins, namely: *Galanthus nivalis* agglutinin (GNA, Terminal Mannose, (1–3), (1–6) or (1–2) linked to Mannose (N- or O-linked glycosylation)), *Sambucus nigra* agglutinin (SNA, Sialic Acid linked (2–6) to Galactose (N- or O-linked glycosylation)), *Maackia amurensis* agglutinin (MAA, Sialic Acid linked (2–3) to Galactose (N- or O-linked glycosylation)), Peanut agglutinin (PNA, Core Disaccharide Galactose (1–3) N-acetylgalactosamine (O-linked glycosylation)), *Datura stramonium* agglutinin (DSA, Gal(1–4)- GlcNAc (N- or O-linked glycosylation) and GlcNAC (O-linked glycosylation)). RSPO1 purification buffer (40 mM Tris-HCl buffer, containing 713 mM NaCl and 5% Trehalose, pH 7.4) was used as a negative control.

The proteins were diluted in 0.1 M sodium carbonate buffer pH 9.6, applied to the ELISA plate and incubated overnight at 4 °C. The plate was washed three times with PBS, blocked for 2 h with Tris-Buffered Saline with 0.05% Tween**®**20 (TBS-T) + 2% polyvinylpyrrolidone (PVP K30 – Sigma Aldrich) at 4 °C and washed 3x again with PBS. For each lectin, the specific lectine-digoxigen coupled was added and incubated 1 h for 4 °C. The plates were washed 3x again. In sequence, the monoclonal anti-digoxigen coupled to alkaline phosphatase antibody (dilution 1:6000) was added and incubated for 1 h at 4 °C. The plate was washed 5x PBS and the BluePhos Microwell Phosphatase Substrate System (KPL) was applied. After 30 min APStop Solution™ was added and the samples were read in a spectrophotometer at 600 nm wavelength (595–650 nm range).

### LC-MS/MS analysis

The sample obtained from the optimized HPLC purification system was subjected to SDS-PAGE and stained with Coomassie blue. The RSPO1 band was distained in water and digested with Glu-C enzyme (Sigma-Aldrich) using the standard protocol for digestion and protein extraction for LC-MS/MS analysis as determined by Aebersold and Goodlet [67]. Samples were subjected to tandem mass spectrometry analysis using the Thermo Scientific FT-ICR Orbitrap LC-MS/MS System (Thermo Fisher Scientific), with an electrospray ionization (ESI) as the ion source, a CID or CAD (y and b ions) fragmentation mode, a FT-ICR/Orbitrap and Linear Ion Trap as the MS and MS/MS scan modes, respectively. Post-translational modifications (PTM) were identified by the mass shift of the peptide fragments in the resulting tandem mass spectrum, which were compared with the PTM theoretical reference mass from the FindMod tool of the Swiss Institute of Bioinformatics platform (ExPASy).

### Statistical analysis

Statistical analysis was performed on GraphPad Prism 6.0, Software Inc., USA. Outliers were removed through the ROUT method. Analysis of Variance (ANOVA) test (Tukey’s post hoc test) and non-parametric student t-test (Mann-Whitney) were used to measure the RSPO1 activity on the in vitro and in vivo assays, respectively. Statistical differences were considered when the *p* < 0.05.

## Supplementary information

**Additional file 1: Figure S1**. Agarose gel fractionation of pUC57/rhR-Spondin1 and pNU1/RSPO1 constructs upon digestion with restriction enzymes. For isolation of the band corresponding to the RSPO1 coding sequence for vector transfer, a sample of pUC57/rhR-Spondin1 was subjected to double digestion with *EcoR*I and *Not*I enzymes and fractionated in a 0.8% agarose gel (A). To confirm the vector exchange, a sample of the pNU1/hRSPO1 construct was subjected to double digestion with the same enzymes and fractionated in 1% agarose gel (B). The undigested constructs were also applied to the gel. MWM: GeneRuler™ DNA Ladder Mix (Thermo Fisher Scientific Inc.). DNA sample mass: 2 μg (A) and 1 μg (B).

**Additional file 2: Figure S2.** First step of rhRSPO1 purification by heparin affinity chromatography. Conditioned medium from an rhRSPO1-overproducing cell clone (Cl. L1) was used for protein purification. A- Chromatogram representing the purification process of rhRSPO1 with a heparin-affinity column in a three step segmented NaCl gradient. The blue line represents the absorbance at the 280 nm UV wavelength and the brown dotted line represents the conductance of the sample. B- Western blot of the purified fractions, detecting the release of the target protein. Samples of the flow through (FT), wash (W) and eluate: Step1 (A1 + A2); Step2 (A4 + A5); Step3 (A7) were used. Equilibration buffer and media conditioned by HEK293 cells transfected with the empty pNU1 vector were used as negative controls (C-Buffer and C-pNU1 Ø, respectively). As positive reference control, the Original Conditioned Medium (OCM) from Cl.L1 cells containing rhRSPO1 was used.

**Additional file 3: Figure S3.** Second step of rhRSPO1 purification by molecular exclusion chromatography. The sample containing rhRSPO1 from step 2 of the heparin affinity column was used in this purification process. The blue line represents the absorbance at the 280 nm UV wavelength and the brown dotted line represents the conductance of the sample. A- Chromatogram representing the purification process of rhRSPO1 using a molecular exclusion column. B- Western Blot of the purification fractions, detecting the release of the target protein into the eluate. Tris-HCl buffer was used as a negative control (C-Buffer), and the Cl.L1 original conditioned media (OCM) and Step2 of the heparin column purification (Step2 Hep) were used as references.

**Additional file 4: Figure S4.** rhRSPO1 in vitro bioactivity. C2C12 cells were treated with two doses of 200 ng/mL of rhRSPO1 obtained from different rhRSPO1-producing cell clones, for up to 5 days. The activity was measured by an alkaline phosphatase (ALP) colorimetric assay. Conditioned medium from HEK293 cells transfected with the empty pNU1 vector (C-) or 293 T cells expressing rhBMP7 were used as negative and positive controls, respectively. ANOVA statistical test (Tukey’s post hoc test) was performed and statistical differences were considered when the *p* < 0.05.

**Additional file 5: Figure S5.** rhRSPO1 glycosylation qualitative profile - Lectin Panel Assay**.** ELISA plate (NUNC-Immuno Plate/ MaxiSorp-NUNC) was coated with 200 ng/μL of purified rhRSPO1 and standard glycoproteins (carboxypeptidase, transferrin, fetuin, asylophetuin), as controls. Specific types of glycan residues in the rhRSPO1 molecule were detected by different lectins, namely: GNA (Terminal Mannose, (1–3), (1–6) or (1–2) linked to Mannose (N- or O-linked glycosylation)); SNA (Sialic Acid linked (2–6) to Galactose (N- or O-linked glycosylation)); MAA (Sialic Acid linked (2–3) to Galactose (N- or O-linked glycosylation)); PNA (Core Disaccharide Galactose (1–3) N-acetylgalactosamine (O-linked glycosylation)); DSA (Gal(1–4)- GlcNAc (N- or O-linked glycosylation) and GlcNAC (O-linked glycosylation)). RSPO1 purification buffer (40 mM Tris-HCl buffer, containing 713 mM NaCl and 5% Trehalose, pH 7.4) was used as a negative control.

**Additional file 6: Figure S6.** LC-MS/MS analysis. The rhRSPO1 sample obtained upon the optimized HPLC purification system was subjected to mass spectrometric analysis (LC-MS/MS) for identification of the glycosylation profile.

## Data Availability

The datasets used and/or analysed during the current study are available from the corresponding author on reasonable request.

## Abbreviations

ALP: Alkaline Phosphatase
Amp^r^: Ampicillin Resistance
ANOVA: Analysis of Variance
Asp: Asparagine
ATCC: American Type Culture Collection
BMP: Bone Morphogenetic Protein
bp: Base pairs
BR: Basic amino acid-rich domain
C-: Negative Control
CAD: Collisionally Activated Dissociation
CD: Crohn Disease
cDNA: Complementary DNA
CDS: Coding Sequence
CHO: Chinese Hamster Ovary
CID: Collision-Induced Dissociation
Cl.: Clone
cm: Centimeter
CMV: Cytomegalovirus
CR-FU: Cysteine-rich furin-like domain
CV: Column Volume
DHFR: Dihydrofolate Reductase
DM: Diabetes Mellitus
DMEM: Dulbecco’s Modified Eagle Medium
DNA: Deoxyribonucleic Acid
DSA: *Datura stramonium agglutinin*
ECM: Extracelular Matrix
ELISA: Enzyme-linked Immunosorbent Assay
ESI: Electrospray Ionization
ExPASy: Swiss Institute of Bioinformatics
FBS: Fetal Bovine Serum
FDA: Food and Drug Administration
Fgf3/4: Fibroblast growth factor
FPLC: Fast Protein Liquid Chromatography
FT: Flow Through
FT-ICR: Fourier-Transform Ion-Cyclotron-Resonance
Fzd: Frizzled
g: Gram; gravity force
GAG: Glycosaminoglycans
GlcNAC: N-Acetylglucosamine
GNA: *Galanthus nivalis agglutinin*
H&E: Hematoxylin and Eosin
HEK: Human Embryonic Kidney
Hep: Heparin
HPLC: High Performance Liquid Chromatography
HSPGs: Heparan Sulfate Proteoglycans
i.v.: Intravenously
IBD: Intestinal Bowel Disease
IRES: Internal Ribosome Entry Site
IU: International Units
kDa: Kilodalton
KRM1: Kremen1
L: Liter
LB: Luria-Bertani; Lysogeny Broth
LC: Liquid Chromatography
Lgr: Leucine Rich Repeat Containing G Protein-Coupled Receptor
LRP: Low-density lipoprotein receptor-related protein
LTR: Long Terminal Repeats
m: Meter
M: Molar
MAA: *Maackia amurensis agglutinin*
mg: Miligram
mL: Mililiter
mM: Milimolar
Mol Excl: Molecular Exclusion
Mo-MuSV: Moloney Murine Sarcoma Virus
MPa: Mega Pascal
mRNA: Messenger RNA
MS: Mass Spectrometry
MWM: Molecular Weight Marker
NCBI: National Center for Biotechnology Information
ng: Nanogram
nm: Nanometer
OCM: Original Conditioned Media
Ori: Origin of Replication
PBS: Phosphate Buffered Saline
PCR: Polymerase Chain Reaction
PNA: *Peanut agglutinin*
PNGase F: N-glycosidase F
PTM: Post-Translational Modifications
rh: Recombinant human
rhRSPO1: Recombinant human Roof plate-specific Spondin
RNA: Rybonucleic Acid
RSPO: Roof plate-specific Spondin
SDS: Sodium Dodecyl Sulfate
SDS-PAGE: Sodium Dodecyl Sulphate-Polyacrylamide Gel Electrophoresis
SFM: Serum Free Media
SHH: Sonic Hedgehog
SNA: *Sambucus nigra* agglutinin
SP: Signal Peptide
TBS: Tris-Buffered Saline
TBS-T: Tris-Buffered Saline + Tween 20
TSR: Thrombospondin type 1 repeat domain
UC: Ulcerative Colitis
Uniprot: Universal Protein Resource
W: Wash
ZNRF3: Zinc and ring finger 3
μg: Microgram
μL: Microliter
μM: Micromolar
