# Supplementary figures and images for: Production, purification and characterization of recombinant human R-spondin1 (RSPO1) protein stably expressed in human HEK293 cells
Source: BMC Biotechnol. 2020 Jan 20;20:5. doi: 10.1186/s12896-020-0600-0 (PMC6971977; doi:10.1186/s12896-020-0600-0)

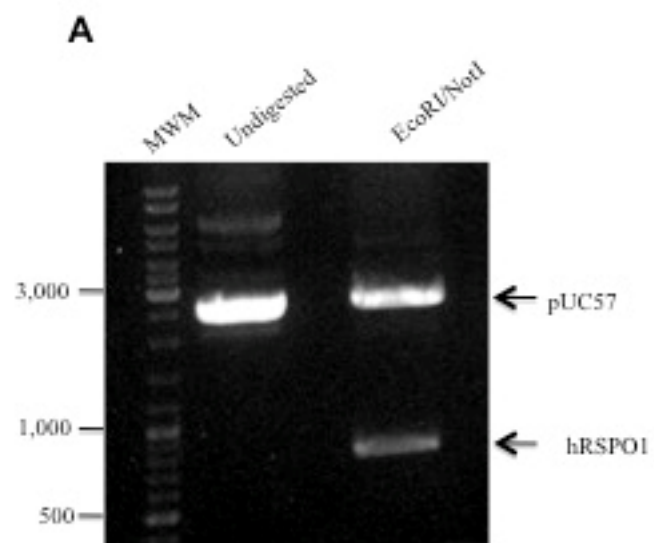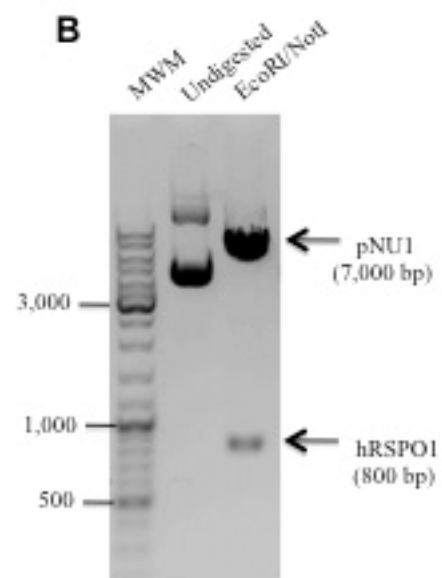

Supplement: Supplementary file 1 — Additional file 1: Figure S1. Agarose gel fractionation of pUC57/rhR-Spondin1 and pNU1/RSPO1 constructs upon digestion with restriction enzymes. For isolation of the band corresponding to the RSPO1 coding sequence for vector transfer, a sample of pUC57/rhR-Spondin1 was subjected to double digestion with EcoRI and NotI enzymes and fractionated in a 0.8% agarose gel (A). To confirm the vector exchange, a sample of the pNU1/hRSPO1 construct was subjected to double digestion with the same enzymes and fractionated in 1% agarose gel (B). The undigested constructs were also applied to the gel. MWM: GeneRuler™ DNA Ladder Mix (Thermo Fisher Scientific Inc.). DNA sample mass: 2 μg (A) and 1 μg (B). [file 12896_2020_600_MOESM1_ESM.pdf]

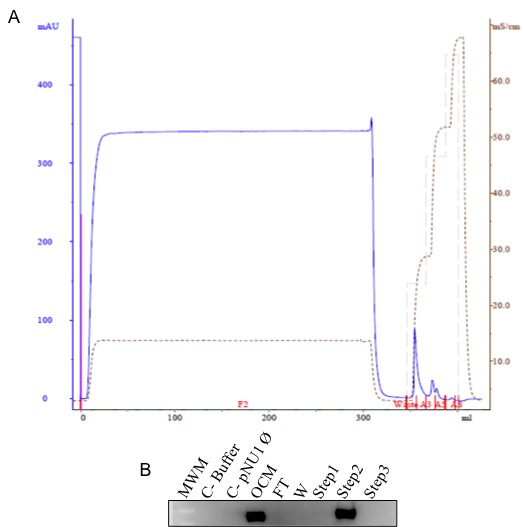

Supplement: Supplementary file 2 — Additional file 2: Figure S2. First step of rhRSPO1 purification by heparin affinity chromatography. Conditioned medium from an rhRSPO1-overproducing cell clone (Cl. L1) was used for protein purification. A- Chromatogram representing the purification process of rhRSPO1 with a heparin-affinity column in a three step segmented NaCl gradient. The blue line represents the absorbance at the 280 nm UV wavelength and the brown dotted line represents the conductance of the sample. B- Western blot of the purified fractions, detecting the release of the target protein. Samples of the flow through (FT), wash (W) and eluate: Step1 (A1 + A2); Step2 (A4 + A5); Step3 (A7) were used. Equilibration buffer and media conditioned by HEK293 cells transfected with the empty pNU1 vector were used as negative controls (C-Buffer and C-pNU1 Ø, respectively). As positive reference control, the Original Conditioned Medium (OCM) from Cl.L1 cells containing rhRSPO1 was used. [file 12896_2020_600_MOESM2_ESM.jpg]

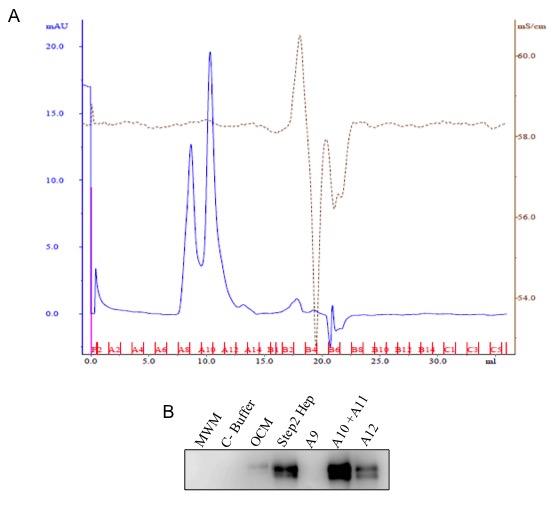

Supplement: Supplementary file 3 — Additional file 3: Figure S3. Second step of rhRSPO1 purification by molecular exclusion chromatography. The sample containing rhRSPO1 from step 2 of the heparin affinity column was used in this purification process. The blue line represents the absorbance at the 280 nm UV wavelength and the brown dotted line represents the conductance of the sample. A- Chromatogram representing the purification process of rhRSPO1 using a molecular exclusion column. B- Western Blot of the purification fractions, detecting the release of the target protein into the eluate. Tris-HCl buffer was used as a negative control (C-Buffer), and the Cl.L1 original conditioned media (OCM) and Step2 of the heparin column purification (Step2 Hep) were used as references. [file 12896_2020_600_MOESM3_ESM.jpg]

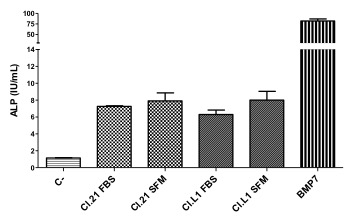

Supplement: Supplementary file 4 — Additional file 4: Figure S4. rhRSPO1 in vitro bioactivity. C2C12 cells were treated with two doses of 200 ng/mL of rhRSPO1 obtained from different rhRSPO1-producing cell clones, for up to 5 days. The activity was measured by an alkaline phosphatase (ALP) colorimetric assay. Conditioned medium from HEK293 cells transfected with the empty pNU1 vector (C-) or 293 T cells expressing rhBMP7 were used as negative and positive controls, respectively. ANOVA statistical test (Tukey’s post hoc test) was performed and statistical differences were considered when the p < 0.05. [file 12896_2020_600_MOESM4_ESM.jpg]

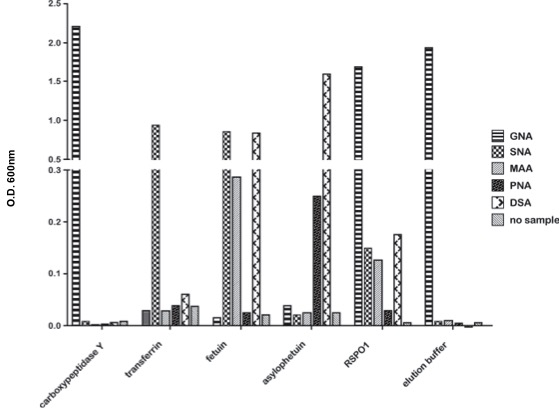

Supplement: Supplementary file 5 — Additional file 5: Figure S5. rhRSPO1 glycosylation qualitative profile - Lectin Panel Assay. ELISA plate (NUNC-Immuno Plate/ MaxiSorp-NUNC) was coated with 200 ng/μL of purified rhRSPO1 and standard glycoproteins (carboxypeptidase, transferrin, fetuin, asylophetuin), as controls. Specific types of glycan residues in the rhRSPO1 molecule were detected by different lectins, namely: GNA (Terminal Mannose, (1–3), (1–6) or (1–2) linked to Mannose (N- or O-linked glycosylation)); SNA (Sialic Acid linked (2–6) to Galactose (N- or O-linked glycosylation)); MAA (Sialic Acid linked (2–3) to Galactose (N- or O-linked glycosylation)); PNA (Core Disaccharide Galactose (1–3) N-acetylgalactosamine (O-linked glycosylation)); DSA (Gal(1–4)- GlcNAc (N- or O-linked glycosylation) and GlcNAC (O-linked glycosylation)). RSPO1 purification buffer (40 mM Tris-HCl buffer, containing 713 mM NaCl and 5% Trehalose, pH 7.4) was used as a negative control. [file 12896_2020_600_MOESM5_ESM.jpg]

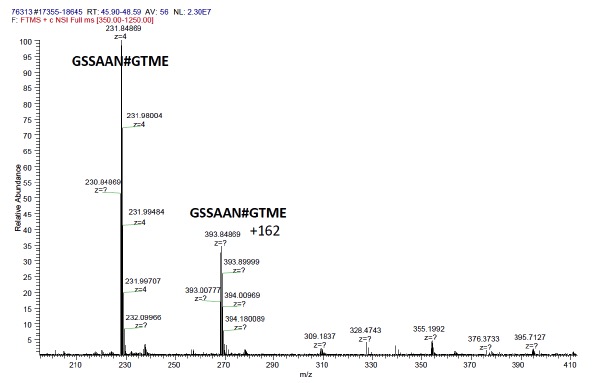

Supplement: Supplementary file 6 — Additional file 6: Figure S6. LC-MS/MS analysis. The rhRSPO1 sample obtained upon the optimized HPLC purification system was subjected to mass spectrometric analysis (LC-MS/MS) for identification of the glycosylation profile. [file 12896_2020_600_MOESM6_ESM.jpg]
